# Optimization of Genotype by Sequencing data for phylogenetic purposes

**DOI:** 10.1016/j.mex.2020.100892

**Published:** 2020-04-20

**Authors:** L.O. Loureiro, M.D. Engstrom, B.K. Lim

**Affiliations:** a University of Toronto, Canada; b Royal Ontario Museum, Canada

**Keywords:** Genotype by Sequencing, Evolutionary relationships, Bats, Molossidae

## Abstract

• Herein we propose a framework for assembling and analyzing Genotype by Sequencing (GBS) data to better understand evolutionary relationships within a group of closely related species using the mastiff bats (*Molossus*) as our model system. Many species within this genus have low-levels of genetic variation within and between morphologically distinct species, and the relationships among them remain unresolved using traditional Sanger sequencing methods. Given that both *de novo* and reference genome pipelines can be used to assemble next generation sequences, and that several tree inference methodologies have been proposed for single nucleotide polymorphism (SNP) data, we test whether different alignments and phylogenetic approaches produce similar results. We also examined how the process of SNP identification and mapping can affect the consistency of the analyses. Different alignments and phylogenetic inferences produced consistent results, supporting the GBS approach for answering evolutionary questions on a macroevolutionary scale when the genetic distance among phenotypically identifiable clades is low. We highlight the importance of exploring the relationships among groups using different assembly assumptions and also distinct phylogenetic inference methods, particularly when addressing phylogenetic questions in genetic and morphologically conservative taxa.

• The method uses the comparison of several filter settings, alignments, and tree inference approaches on Genotype by Sequencing data.

• Consistent results were found among several approaches.

• The methodology successfully recovered well supported species boundaries and phylogenetic relationships among species of mastiff bats not hypothesized by previous methods.

## Graphical abstract

**Figure:**
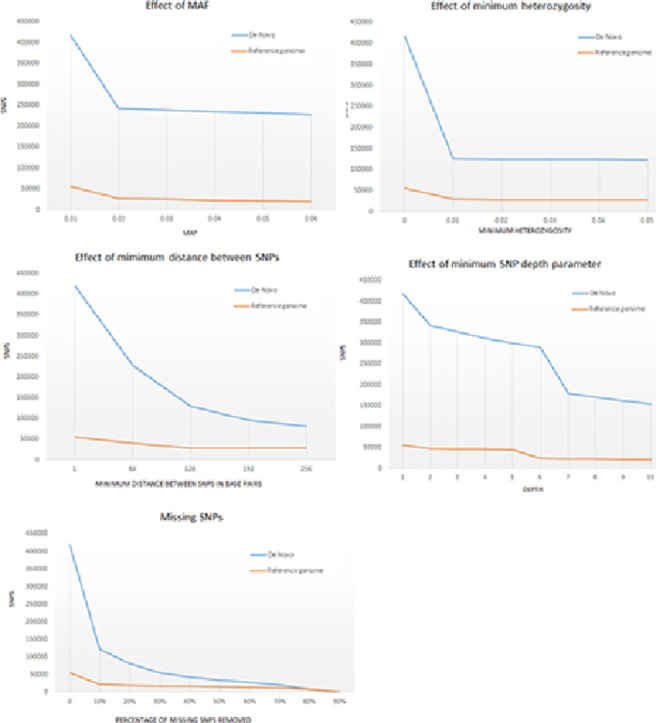

Specification Table

**Table:** 

Subject Area:	Biochemistry, Genetics and Molecular Biology
More specific subject area:	*Evolution, phylogeny, genetics*
Method name:	Genotype by Sequencing phylogenetic analyses optimization
Name and reference of original method:	*NA*
Resource availability:	*NA*

## *Method details

### Background

Advances in genomics technology have allowed the generation of large numbers of molecular markers across the genome, which increases sample sizes and provides additional data to help resolve interpretation of the ecology and evolution of traditionally poorly understood species groups [1]. One of these methods is Genotyping by Sequencing (GBS), which involves sequencing genomic regions flanking restriction sites. Using GBS, many sequences of short length are obtained, vastly increasing the size of the overall data set in comparison to traditional Sanger methods. This technique provides sequence data for thousands of single nucleotide polymorphisms (SNPs), allowing the detection of small, but consistent genetic variation among genetically similar groups not revealed by standard gene sequencing approaches. GBS has been successfully used in studies of population genetics [2], phylogenetic analysis [3], [4] and phylogeography [5], [6].

In this context, we propose a framework for assembling and analyzing GBS data to better understand evolutionary relationships among species of mastiff bats (*Molossus*), a genus with a complex taxonomic history and low levels of genetic variation [7]. Herein, we test how four different filtering settings affect the accuracy and consistency of our data. Given that both *de novo* and reference genome pipelines are often used to assemble next generation sequencing (NGS) data, and that several tree inference methodologies have been proposed for SNP data, we also test if different alignments and phylogenetic approaches produce similar results. These data offer a useful framework for other comparative studies of ecology and evolution using the GBS approach.

### Methodology

We obtained tissues from a total of 189 specimens including all the currently recognized species of *Molossus* [8] and representatives of two other species of molossid bats, *Promops centralis* and *Eumops auripendulus*, that were used as outgroups following Ammerman et al. [9] and Gregorin and Cirranello (2016) [10]. Genomic DNA was isolated using a Qiagen DNeasy extraction kit (Qiagen, Inc. Valencia, CA, USA) following the manufacturer's instructions. Genomic DNA quality was checked by visual inspection on agarose gels and quantified using a Nanodrop spectrophotometer (Nanodrop Technologies). Thirty microlitres of high quality (>100 ng/ul) DNA per sample was used for the library preparation. We submitted the samples to the Cornell Institute of Genomic Diversity (IGD) to obtain SNP datasets through the GBS approach following the protocol described by Elshire et al. [11]. All libraries were sequenced on an Illumina HiSeq 2000.

**Fig. 1:**
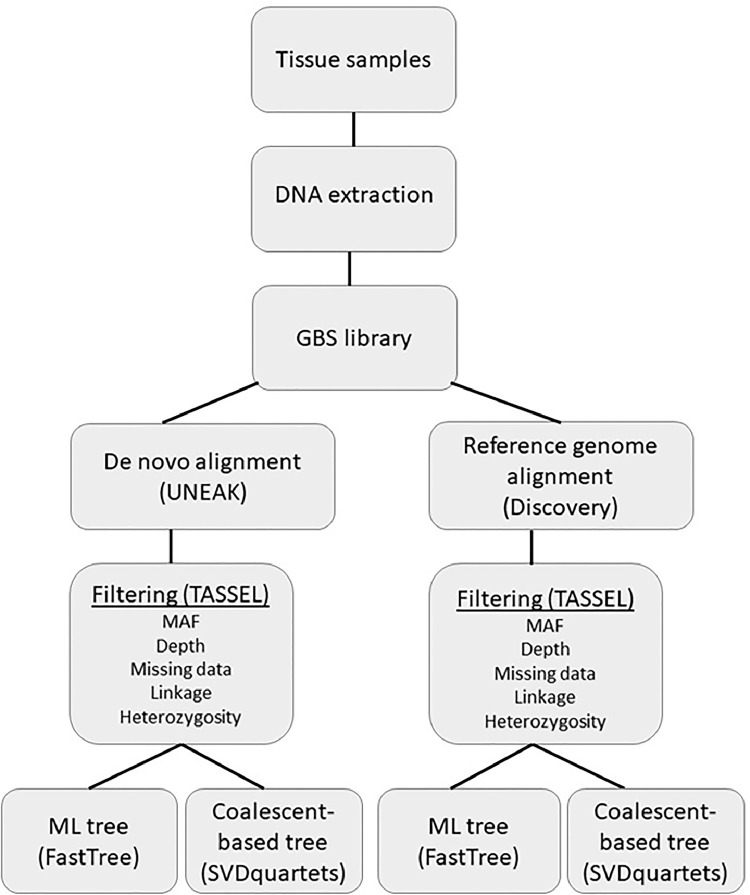
Flow chart of the different methodologies used in the analyses. Acronyms are discussed in the text.

Two different approaches were used to process the data and test the accuracy and precision of the results. Raw sequence files from Illumina were converted into individual genotypes using the Discovery and the Universal Network-Enabled Analysis Kit (UNEAK) pipelines, available as part of the TASSEL 3.0 software [12] (Fig. 1). Both pipelines trim the sequences to a length of 64 basepairs (bp) after the barcode and discard shorter reads. Identical reads are clustered into tags, and all identical tags are merged. The Discovery pipeline uses a reference genome to align the tags [13], and for this alignment we used the genome of *Myotis brandtii* in the related family Vespertilionidae [14], [15]. Unfortunately, there were no genomes available for the family Molossidae in the time of the analyses. The UNEAK pipeline uses a *de novo* approach, and the alignment to the reference genome is replaced with a pairwise alignment of tags, in which tag pairs with a 1 bp mismatch are considered as candidate SNPs [16]. The *de novo* alignment assumes homogeneity of rates across sequenced tags [17] and although it has been proven to produce highly supported trees (Rojas et al., 2003; Wagner et al., 2012), there is still a potential source of inaccuracy in the homology assumption that is determined based on the similarity between tags. The sequences produced by this method are short (64 bp in the GBS approach), and without a reference genome, sequences from different individuals might be mistakenly assigned as homologous given their base similarity. The use of the reference genome is expected to decrease errors in the inference of orthologous sequence because it allows genomic mapping of loci, and partitioning analysis by linkage groups, coding and non-coding positions, and other genetic subregions, all of which could potentially improve phylogenetic accuracy [17], [18]. Unfortunately, a clear limitation in our study is that no genome was available for any species of Molossidae in the time of the analyses. To address this bias, we examined the data generated using a reference genome and a *de novo* alignment separately.

We assessed the quality of the paired GBS-tags using FastQC. The likelihoods of the possible genotypes were estimated for both pipelines, and the Genotype quality (GQ), which is the difference between the most likely and the second most likely genotype, was calculated also using FastQC. The data were then imported into TASSEL where samples were demultiplexed and filtered. The amount of missing data acceptable for phylogenetic estimates still lacks a consensus. The identification of minimum values for these filters is still debatable and may ultimately depend on the dataset in hand and the question that needs to be answered [18], [19], [22]. Some studies have been very conservative, removing all loci that are missing for any taxon in the dataset (McCormack et al., 2012; [20]), but others included loci that were not present in a large proportion of samples [5], [6]. The amount of missing data allowed in the SNPs matrix can affect further analyses, however, some authors argue that larger matrices, even those containing a large amount of missing data, may provide greater phylogenetic accuracy than smaller ones [3], [5], [19]. Therefore, we tested if the amount of missing data removed would affect our final analyses.

Similarly, the optimum value for minimum allele frequency (MAF) is not universally agreed upon and there is a trade-off between the use of MAF and the loss of rare alleles. The increase of the MAF value may cause an under-calling of heterozygotes with the loss of biological information, instead of the removal of sequencing errors [21]. Kim et al., [22] argued that for rare SNPs (e. g. MAF <0.01) differentiation between sequencing errors and true rare alleles is difficult, and they should be discarded. Lincky and Battey (2019) [23] showed that highly accurate population inferences are reached when rare alleles are included (MAF 2% to 8%), but decay in accuracy when only common alleles were included. Here, we tested how different MAF values varying from 0.01 to 0.06 would affect the number of SNPS in our data sets and the accuracy and consistency of estimation of phylogenetic relationships.

Low depth sequences are sequences recovered by relatively few reads and, if not removed, they might lead to serious bias (2014) [24]. Sequences with very low depths might increase the probability of calling false SNPs due to PCR and sequencing errors and must be removed [25]. However, excess removal might also reduce the amount of informative DNA sequences [26]. In our data set, we first estimated the mean depth for the reference and *de novo* alignments using VCFtools, and then we simulated how the depth could affect the number of SNPs in both alignments by removing sequences with lower and higher depths than the mean in the TASSEL software. For this simulation we looked at the number of SNPs removed and the agreement between final topologies using the optimum MAF value found for each dataset.

GBS sequences are short, which decreases the chance of intragenic recombination. However, if the SNPs are closely linked on the same chromosome, they might not be independent. Some studies suggest mapping the SNPs in a reference genome to confront this issue [27], [28]. The lack of an available closely related reference genome to *Molossus* and the low percentage of tags that aligned to the *Myotis* genome (2.5%), precluded use of this method for our dataset. Each tag produced by the GBS method has 64 bp, and therefore we have tried to overcome this problem by removing SNPs that were separated by less than 128 bp in the genome.

To test for divergence in tree inference approaches we removed invariant sites in both alignments and reconstructed the phylogenetic relationships within the genus *Molossus* using the Maximum Likelihood approach (ML) implemented in FastTree [29] with a GTR + gamma model of nucleotide evolution estimated by Partition Finder 1.0.1 [30] (Fig. 1). In the ML method, the alignment for each locus is aggregated in a supermatrix and a species tree is estimated under the assumption that all sites evolve identically and independently [31]. The relationships among clades were also investigated through a coalescence approach, which accounts for differences in genealogical histories of individual loci using the program SVDquartets [32] implemented in PAUP 4.0 [33] (Fig. 1). The SVDquartets is a coalescent model that uses unlinked SNPs to infer the quartet tree for every four species, and then combines all the subtrees into a species tree [32]. Pettengill et al. [18] found that some programs produce more reliable trees for NGS, even when working with the same phylogenetic assumptions (e.g. ML), but the difference in topologies is usually in poorly supported nodes. Four independent runs were conducted to access topological convergence, each including 500 bootstrap replicates and exhaustive quartet sampling.

### Method validation

After merging the Illumina paired-end sequences, more than nine million tags remained. The *de novo* pipeline identified 418,810 SNPs after error curation in the standard network error remover of TASSEL. The alignment using the reference genome discarded 96.6% of tags that did not align with the *Myotis* genome and 0.9% of the tags that aligned multiple times, keeping only 2.5% of the short length sequences. After removal of invariant sites, the reference genome alignment produced 55,350 SNPs. Of the 189 specimens included in the Illumina library, 23 were discarded in the *de novo* pipeline and 16 were discarded in the reference-based pipeline because they had low numbers of raw reads and few loci (>90% of missing data), probably because of low DNA quality. The quality of the isolated DNA depends on the quality of the tissue sample. Fresh tissues produce the highest DNA yield and quality and samples should be stored under conditions that preserve DNA integrity. In addition, repeated freezing and thawing of frozen tissues might reduce the size and quality of DNA. For compromised samples, the initial concentration was lower than required by GBS library preparation (>100 ng/ul), and multiple DNA concentration procedures were required to archive the required DNA quality. After this procedure, thirty microlitres of each sample were sent to sequence, but the remaining DNA aliquot were less than thirty microlitres per individual, too low for repeating the GBS library preparation.

**Fig. 2:**
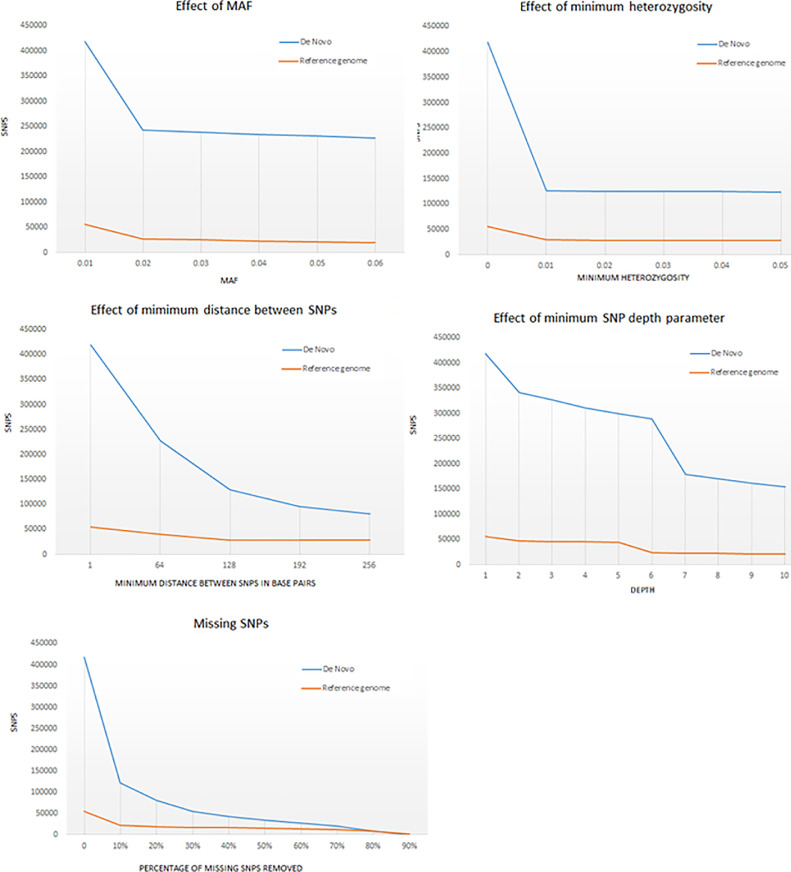
Comparison of different parameters in the number of SNPs obtained with the *de novo* alignment and the reference genome alignment for *Molossus*.

Both alignments behaved similarly for all filters. In our dataset, the removal of 10% missing SNPs decreased the total number of markers by less than half. The number of SNPs decreased further when removing 20% to 90% of missing data (Fig. 2). Our final trees did not change when more than 50% of missing data was removed, but loss of support was observed in some clades when allowing for missing data higher than 50%. Therefore, if the SNPs were missing in 50 % or less of the samples, we kept the marker, but if the marker was present in less than 50% of the samples they were discarded.

Minor alleles are expected to occur in a smaller percentage of the dataset, usually in less than 10% of the population [21], [22], [34], [35]. Our simulations showed that the number of SNPs removed with MAF lower than 2% did not affect our the number of SNPs significantly. For either alignments, only a small number of SNPs was discarded (<2% for both pipelines) with MAF >0.02, which is consistent with true polymorphic sites. However, large parts of the dataset were excluded with the removal of MAF < 0.02 (58% with the *de novo* and 49% with the reference genome approaches) (Fig. 2), which is more likely to represent sequence errors.

Variation in DNA quality can affect genotype accuracy. Low DNA quality, or concentration, often affect SNPs identification, and the genotype heterozygosity rate can be used as a measure of the accuracy of the samples [36]. In our dataset, the same pattern found for MAF occurred for the heterozygosity filter, but the heterozygosity filter had a higher impact in the *de novo* alignment in comparison with the reference genome data set. When set to 0.01, this filter discarded approximately two-thirds of the SNPS in the *de novo* alignment and half of those in the reference genome alignment (Fig. 2).

More than half of the SNPS were closely aligned in the genome, which could indicate linked loci. We removed SNPs that were 128 base pairs apart, twice the length of a single tag, to decrease linked SNPs in the dataset (Fig. 2). Linkage analyses indicate that only nine pairs of linked SNPs remained after the removal of SNPs 128 bp apart in the de novo alignment and only two pairs in the reference genome alignment, which were removed manually for further analyses. Although the removal of SNPs with distances higher than 128 bp apart discarded a larger percentage of potentially linked SNPs, the increase of this filter value also removed more than 34,000 non linked SNPs in the *de novo* alignment and more than 300 in the reference genome alignment. The removal of linked SNPs did not affect the other parameters of the data sets (e. g. mean depth) and species relationships compared to the linked dataset. However, the relationships within some populations changed slightly, which might indicate a higher effect on population structure analyses.

The average depth in the *de novo* and reference genome alignments were seven and six respectively, which is consistent with our analysis that shows a plateau in the number of SNPs after setting missing data depth less than six and seven for each respective data set (Fig. 2). The depths were calculated using the best parameter for MAF (0.02) in VCFtools. For both *de novo* and reference genome pipelines, the removal of sequences with less than the mean depth caused a considerable decrease in SNPs (44% in the *de novo* and 43% in the reference genome pipeline), and this high proportion of low depth tags suggests a high degree of uncertainty about the homology of those sequences [18]. However, when sequences with depth higher than the average were set as missing data, the number of SNPs included was not greatly affected but the removal of those sequences led to the loss of rare variants. Indeed, when values larger than the mean depth were removed, de novo and reference genome topologies lost support for some clades, but branching sequences were not changed. In the final analyses, only tags with lower depth than the mean were removed.

According to the previous results, the sequences were filtered for missing data < 50%, minor allele frequency (MAF) >0.02, heterozygosity >0.01, and depth coverage lower than seven and six for the *de novo* and reference alignment, respectively. After data filtering, the *de novo* pipeline yielded 71,801 SNPs and the reference genome pipeline yielded 27,323 SNPs. To remove unlinked and uncertain SNPs, sites with more than 50% of missing data were discarded as were sites less than 128 bp apart. The final data set with unlinked SNPs had 29,448 SNPs for the *de novo* pipeline and 15,569 SNPs for the reference genome dataset. In addition, we also used more and less conservative filtering values to test for differences in accuracy and consistency in tree topologies.

### Validation results

The ML and SVDquartets trees with both the *de novo* and the reference genome alignments are congruent when the optimum filtering settings are used, recovering the same clades and the same relationships among species [7], [8]. However, when more conservative filter values, compared to those optimum values found in previous analysis, are used (e. g. MAF>0.02; heterozygosity >0.01), clades within *Molossus* start to lose support; although, the relationships among them do not change. This outcome is consistent with loss of true rare polymorphic sites, which should not be removed from the dataset. The removal of markers with less than 50% of missing data did not change supports in the phylogenetic trees. However, if markers with more than 50% of missing data are removed and less conservative filtering setting values are used (MAF<0.02; heterozygosity <0.01), the topologies between de novo and reference genome alignment lose agreement and relationship among some clades within *Molossus* are no longer supported by both alignments. These results are consistent with incorporation of sequencing errors. Using the optimum filtering values (Fig. 2), the best scoring ML and SVDquartets trees contained most nodes with 100% bootstrap support, and a few nodes with lower support, which were always above 80% [7], [8]. Although both alignment approaches produced the same species-level trees, there were minor shifts in relationships within well supported clades at a population level [7], [8]. Although the two inference methods used in this study have different assumptions and work with different algorithms, we still recovered the same topology at the species level, supporting the assessment of phylogenetic relationships [7], [8].

The greater power of next-generation sequencing approaches compared to Sanger methods for answering phylogenetic questions is well established [37], [38], but there are only a few studies comparing the concordance between *de novo* and reference-based alignments. In our study, relationships within terminal clades with low bootstrap support were affected by the choice of the pipeline, which could have resulted from the difference in number of SNPs retained from both alignments. However, the use of the reference genome in the alignment does not seem to be essential for recovering a robust overall phylogenetic tree, since both phylogenies, *de novo* and with the reference genome, were similar at the species level when optimal filtering settings values were used.

We highlight the importance of exploring the relationships among groups using different assembly assumptions and also distinct phylogenetic inference methods, particularly when addressing phylogenetically conservative groups. All models of molecular evolution are a simplification of the actual evolutionary process, and the inappropriate choice of filters during alignment or tree inference can lead to systematic bias in the phylogenetic reconstruction [39], [40]. Therefore, we emphasize the value of carefully optimizing SNPs filters to minimize the effect that missing data, independent SNPs, and incorrect inference of homology could have on the results.
